# Evaluation of Drug-Related Problems in Chronic Kidney Disease Patients

**DOI:** 10.7759/cureus.24019

**Published:** 2022-04-10

**Authors:** Tasneem M Shouqair, Syed Arman Rabbani, Sathvik B Sridhar, Martin T Kurian

**Affiliations:** 1 Department of Clinical Pharmacy and Pharmacology, Ras Al Khaimah College of Pharmacy, Ras Al Khaimah Medical and Health Sciences University, Ras Al Khaimah, ARE; 2 Department of Nephrology, Ibrahim Bin Hamad Obaidullah Hospital, Ras Al Khaimah, ARE

**Keywords:** adverse drug reactions, potential drug-drug interactions, drug-related problems, polypharmacy, chronic kidney disease

## Abstract

Background

Chronic kidney disease (CKD) is a challenging global health problem with increasing prevalence worldwide. Concurrence of CKD and comorbidities results in the use of multiple medications and exposing patients to polypharmacy. Polypharmacy in CKD is common across all the stages of the disease and leads to poor medication adherence, higher healthcare costs, and drug-related problems, such as drug-drug interactions (DDIs) and adverse drug reactions (ADRs). DDIs and ADRs in CKD patients may lower the quality of life, increase the length of hospital stay, and augment the risks of morbidity and mortality.

Methodology

This was a hospital-based, prospective, cross-sectional study conducted in a secondary care hospital. The study population comprised 130 adult CKD patients admitted to the nephrology department including those on maintenance hemodialysis. Study-related data were obtained from the electronic patient case records. Medications prescribed to the patients were analyzed for potential DDIs (pDDIs) using Portable Emergency and Primary Care Information Database (PEPID 12.1) drug interaction checker. All observed and reported suspected ADRs related to the prescribed drugs were evaluated for causality, severity, preventability, and predictability.

Results

Out of the 130 patients, majority were males (n = 71, 54.6%), in the age group of 61-70 years (n = 45, 34.6%), and belonged to CKD stage 5 (n = 105, 80.8%). The mean number of drugs prescribed was 11.1 ± 3.8 per patient. The prevalence of pDDIs was found to be 89.2%. Upon analysis by the PEPID database, 708 pDDIs with 215 different pairs of interacting drugs were identified. Polypharmacy (odds ratio (OR): 62.34, 95% confidence interval (CI): 7.97-487.64, p < 0.001) was identified as an independent predictor of the occurrence of pDDIs. Negative binomial regression analysis revealed that dyslipidemia (incidence rate ratio (IRR): 2.7, 95% CI 2.09-3.48, p < 0.001) and diabetes (IRR: 1.2, 95% CI 1.01-1.54, p = 0.040) increased the probability of occurrence of pDDI by 2.7 and 1.2 folds, respectively. Furthermore, the likelihood of pDDI increased with every one-day increase in the length of hospital stay (IRR: 1.02, 95% CI 1.00-1.03, p = 0.015) by 1.02 times and polypharmacy (IRR: 6.30, 95% CI 3.04-13.02, p < 0.001) by 6.3 times. The incidence of ADRs was found to be 10.7%. Majority of suspected ADRs were possible (n = 7, 50.0%), of mild and moderate severity (n = 7, 50.0%), and non-preventable (n = 8, 57.1%) type.

Conclusions

This study investigated two important drug-related problems, pDDIs, and ADRs, in the CKD population. High proportion of CKD patients in the study had pDDIs. Comorbid conditions such as dyslipidemia and diabetes mellitus, length of hospital stay, and polypharmacy were significantly associated with increased likelihood of pDDIs. Furthermore, there was a burden of ADRs in the study population, of which most ADRs were possible and of mild to moderate severity. Prevention, identification, and resolution of these problems in CKD patients is important and can be achieved through medication optimization, which requires a proactive interdisciplinary collaboration between clinicians, clinical pharmacists, and other healthcare professionals.

## Introduction

Chronic kidney disease (CKD) is a challenging global health problem with growing prevalence worldwide and is linked to increased morbidity, mortality, and healthcare expenditures [1]. CKD is associated with several complications affecting different organ systems and patients with CKD experience poor quality of life [2]. Concurrence of CKD and comorbidities results in the use of multiple medications making the management of CKD particularly challenging [3]. Polypharmacy, the concomitant use of five or more medications, is a significant and rising public health concern [4]. Polypharmacy in CKD is common across all the stages of the disease and is linked to poor medication adherence, higher healthcare costs, and, most significantly, drug-related problems, such as drug-drug interactions (DDIs) and adverse drug reactions (ADRs) [5,6].

DDIs are changes that occur in the effects of one drug due to the concomitant administration of another drug for the same or different diseases and can be a result of pharmacokinetic or pharmacodynamic alterations [7]. DDIs account for 2-6% of all hospital admissions and have been implicated in adverse clinical outcomes. The estimated prevalence of DDIs in CKD patients ranges from 56.9% and 89.1% [8]. Early identification of potential DDIs (pDDIs) is vital to implement appropriate preventive measures and interventions [8]. Furthermore, ADRs are prevalent, frequently serious, and potentially preventable in CKD patients [9]. Therefore, assessing the causality, severity, and preventability of ADRs is essential to reduce the likelihood of associated complications [10].

DDIs and ADRs in CKD patients may lower the quality of life, increase the length of hospital stay and augment the risks of morbidity and mortality [11]. Therefore, prevention, identification, and resolution of these problems are very important for this vulnerable CKD population, which, in turn, will lead to better clinical outcomes. This can be achieved through medication optimization that requires a proactive interdisciplinary collaboration between clinicians, clinical pharmacists, and other healthcare professionals [12].

Evaluation of drug-related problems such as pDDIs and ADRs among CKD population of United Arab Emirates (UAE) is important for several reasons, increasing the burden of the disease in the local population [13], largely unknown state of CKD care in the region, and paucity of studies addressing drug-related problems in its CKD population. Therefore, the present study was undertaken to assess pDDIs and ADRs among CKD patients presenting to the nephrology department of a secondary hospital in the UAE.

## Materials and methods

Study setting and population

The study was conducted at Ibrahim Bin Hamad Obaidullah Hospital, which is a secondary care hospital in Ras Al Khaimah, UAE. The hospital is a multispecialty center with 167 beds and specialties such as internal medicine, nephrology, cardiology, neurology, emergency medicine, geriatrics, and psychiatry. The study population included adult CKD patients admitted to the nephrology department including patients on maintenance hemodialysis at the study site.

Study design and sample size

This was a hospital-based, prospective, cross-sectional study. A priori statistical sample size calculation was performed using Epi Info™ software version 7.2 by the Centers for Disease Control and Prevention (CDC). Single population proportion formula was used to estimate the sample size using CKD prevalence in the region 4.6% [14], with a 4% margin of error at 95% confidence interval (CI) level and design effect of 1.0, the estimated sample size came out to be 105 patients. A total of 130 CKD patients were included in the study.

Variable definitions

CKD and stages of CKD were defined as per the Kidney Disease: Improving Global Outcomes (KDIGO) clinical practice guideline for the evaluation and management of CKD [15]. Diabetes mellitus was defined as the use of antidiabetic drugs or HbA1c of ≥6.5%. Anemia was defined as hemoglobin of <13 g/dL in males and <12 g/dL in females. Hypertension was defined as the use of antihypertensive drugs or systolic blood pressure of ≥140 mmHg and/or diastolic blood pressure of ≥90 mmHg. Dyslipidemia was defined as the use of lipid-lowering medications or total cholesterol of ≥5.2 mmol/L and/or triglycerides of >1.7 mmol/L and low-density lipoprotein cholesterol of >2.6 mmol/L. Hyperphosphatemia was defined as the use of phosphate binders or serum phosphate level of >1.45 mmol/L. Vitamin D deficiency was defined as the use of vitamin D preparations or serum 25-hydroxyvitamin D level of <50 nmol/L. Polypharmacy was defined as using five or more drugs concomitantly.

Data collection and analysis

Study data were obtained from the electronic patient case records by the investigators. The collected data included demographic, clinical, and laboratory data. The drugs prescribed to the patients were recorded and analyzed for pDDIs using Portable Emergency and Primary Care Information Database (PEPID 12.1) drug interaction checker [16]. The identified pDDIs were characterized as pharmacokinetic (PK), pharmacodynamic (PD), or other based on the mechanism and graded as non-significant, minor, moderate, significant, or life-threatening based on the level of concern [16]. All observed and reported suspected ADRs related to the prescribed drugs were documented and evaluated for causality [17,18], severity [19], preventability [20], and predictability [20].

Data were reviewed for completeness, sorted, and analyzed using Statistical Package for the Social Sciences (SPSS) version 26.0 (IBM Corp., Armonk, NY, USA). Descriptive statistics were used to analyze the study variables and the results were reported as frequency, mean, and standard deviation (SD) with 95% CIs. Univariate and multivariate logistic regression analyses were performed to identify the predictors of pDDIs. The association between the number of pDDIs and CKD patients’ variables was established using a negative binomial regression model and results were reported as incidence rate ratios (IRR) with 95% CI that determined the strength of associations. P-values of <0.05 were considered statistically significant.

Ethical considerations

Ethical approval was obtained from the Institutional Ethics Committee of Ras Al Khaimah (RAK) Medical and Health Sciences University (RAKMHSU-REC-016-2019-PG-P) and the Ministry of Health and Prevention Research Ethics Committee/RAK Subcommittee (MOHAP/REC/2019/44-2019-PG-P), UAE.

## Results

Demographic and clinical characteristics

Of the 130 patients enrolled in the study, majority were males (n = 71, 54.6%), belonged to the age group of 61-70 years (n = 45, 34.6%), were non-smokers (n = 127, 97.7%) and non-alcoholic (n = 130, 100%), with no previous allergic history (n = 116, 89.2%), and belonged to CKD stage 5 (n = 105, 80.8%). The majority of the patients had hypertension as the comorbid condition (n = 126, 96.9%). The number of associated comorbidities varied between two to eight with a mean number of 5.5 ± 1.3 comorbidities. Hospital stay varied among patients from a minimum of one day to a maximum of 25 days with an average length of hospital stay of 3.9 ± 5.7 days. In total, 1,494 drugs were administered to 130 CKD patients. The mean number of drugs administered was 11.1 ± 3.8 per patient. The majority of the patients (n = 116, 89.2%,) were prescribed more than five drugs. The demographic and clinical characteristics are represented in Table 1.

**Table 1:** Demographic, clinical, and laboratory characteristics of CKD patients. ^a^Cardiovascular disease is defined as myocardial infarction, cardiac arrhythmia, heart failure, peripheral artery disease, or stroke. ^b^Neurological disease is defined as epilepsy, Alzheimer’s disease, depression, schizophrenia, or anxiety. ^c^Polypharmacy is defined as intake of five or more medications per day SD: standard deviation; CI: confidence interval; CKD: chronic kidney disease; RBG: random blood glucose

Variables		N (%)/ Mean ± SD	95% CI
Gender, n (%)	Female	59 (45.4)	36.9-53.8
	Male	71 (54.6)	46.2-63.1
Age (years), n (%)	18–30	7 (5.4)	1.5-10
	31–40	9 (6.9)	3.1-12.3
	41–50	18 (13.8)	8.5-20
	51–60	23 (17.7)	11.5-24.6
	61–70	45 (34.6)	25.4-42.3
	71–80	22 (16.9)	10.8-23.8
	81–90	6 (4.6)	1.5-8.5
Nationality, n (%)	Arab	109 (83.8)	76.9-90
	Non-Arab	21 (16.2)	10.0-23.1
Tobacco use, n (%)	Yes	3 (2.3)	0.0-5.4
	No	127 (97.7)	94.6-100
Alcohol use, n (%)	No	130 (100)	100-100
Previous allergic history, n (%)	Yes	14 (10.8)	6.2-16.9
	No	116 (89.2)	83.1-93.8
CKD stage, n (%)	Stage 1	0.0	0.0
	Stage 2	2 (1.5)	0-3.8
	Stage 3	10 (7.7)	3.1-12.3
	Stage 4	11 (8.5)	3.8-13.1
	Stage 5	107 (82.3)	76.2-88.5
Number of comorbidities, n (%)	≤4	20 (15.4)	9.2-22.3
	>4	110 (84.6)	77.7-90.8
Type of comorbidities, n (%)	Hypertension	126 (96.9)	93.1-99.2
	Hyperphosphatemia	109 (83.8)	79.9-89.2
	Anemia	108 (83.1)	76.2-89.2
	Diabetes mellitus	92 (70.8)	63.1-77.7
	Dyslipidemia	88 (67.7)	58.5-75.4
	Vitamin D deficiency	75 (57.7)	48.5-66.2
	Secondary Hyperparathyroidism	53 (40.8)	32.3-50
	Cardiovascular diseases^a^	43 (33.1)	25.4-41.5
	Neurological diseases^b^	19 (14.6)	9.2-20.8
Polypharmacy^c^, n (%)	<5 drugs	14 (10.8)	5.4-16.2
	≥5 drugs	116 (89.2)	83.8-94.6
Length of hospital stay (days), n (%)	<7 days	104 (80.0)	73.1-86.2
	≥7 days	26 (20.0)	13.8-26.9
Systolic blood pressure (mmHg), mean ± SD		150.1 ± 22.1	146.3 ± 19.6-153.9 ± 24.5
Diastolic blood pressure (mmHg), mean ± SD		76.1 ± 14.5	73.6 ± 12.6-78.7 ± 16.2
Hemoglobin (g/dL)		10.8 ± 1.6	10.6 ± 1.3-11.1 ± 1.8
Serum creatinine (mmol/L)		654.0 ± 349.1	594.1 ± 268.8-718.1 ± 438.7
Urea (mmol/L)		19.8 ± 9.3	18.2 ± 7.8-21.5 ± 10.8
Serum phosphorus (mmol/L)		1.3 ± 0.6	1.2 ± 0.5-1.4 ± 0.7
Serum calcium (mmol/L)		2.0 ± 0.2	2.0 ± 0.1-2.1 ± 0.2
Serum potassium (mmol/L)		4.5 ± 0.9	4.3 ± 0.7-4.6 ± 1.0
Serum sodium (mmol/L)		137.5 ± 3.2	136.9 ± 2.8-138.0 ± 3.6
RBG (mmol/l)		8.0 ± 4.1	7.4 ± 3.2-8.7 ± 5.1
HbA1c (%)		5.5 ± 1.1	5.4 ± 0.8-5.7 ± 1.2

Drug-related problems

Potential Drug-Drug Interactions

**Figure 1:**
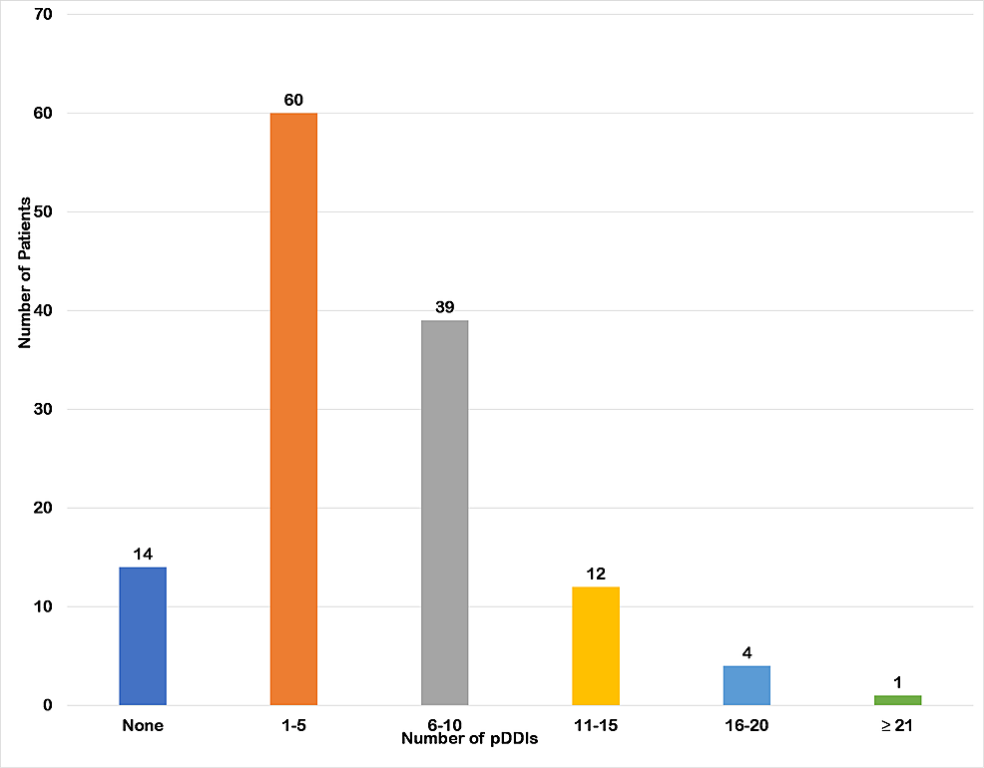
Distribution of potential drug-drug interactions. pDDIs: potential drug-drug interactions

The prevalence of pDDIs in our study was 89.2% (116 patients had pDDIs). Upon analysis using the PEPID database, a total of 708 pDDIs with 215 different pairs of interacting drugs were identified in 116 patients. The mean pDDIs per patient was found to be 5.4 ± 4.4. The majority of the patients had (n = 60, 46.2%) had 1-5 interactions, followed by 6-10 interactions (n = 39, 30.0%). Eight interactions were life-threatening, 55 interactions were significant, and 152 interactions were moderate according to the level of concern. The common pDDIs reported were between aspirin and enoxaparin (n = 30, 23.1%), insulin and linagliptin (n = 22, 16.9%), moxonidine and bisoprolol (n = 14, 10.8%), clopidogrel and enoxaparin (n = 13, 10.0%), linagliptin and gliclazide (n = 8, 6.2%), and aspirin and urokinase (n = 6, 4.6%). Table 2 represents the common pDDIs detected in the study. Figure 1 represents the distribution of pDDIs in the study.

**Table 2:** Common potential drug-drug interactions reported in the study. pDDI: potential drug-drug interactions; Other: interaction type other than pharmacokinetic or pharmacodynamic

pDDIs	Percentage	Interaction types	Pharmacological consequences
Aspirin-enoxaparin	23.0	Pharmacodynamic	Aspirin may enhance the anticoagulant effect of enoxaparin. Increased possibility of bleeding
Insulin-linagliptin	16.9	Other	Linagliptin may enhance the hypoglycemic effect of insulin
Moxonidine-bisoprolol	10.7	Other	Moxonidine may enhance AV-blocking effect of bisoprolol. Significance of this interaction may be more in heart failure patients
Clopidogrel-enoxaparin	10.0	Pharmacodynamic	Clopidogrel may enhance the anticoagulant effect of enoxaparin. Increased possibility of bleeding
Linagliptin-gliclazide	6.1	Other	Linagliptin may enhance the hypoglycemic effect of gliclazide
Aspirin-urokinase	4.6	Pharmacodynamic	Aspirin may enhance the anticoagulant effect of urokinase. Increased possibility of bleeding
Sevelamer-levothyroxine	3.8	Other	Sevelamer may decrease the serum concentration of levothyroxine
Carvedilol-cinacalcet	3.0	Pharmacokinetic	Cinacalcet may increase the serum concentration of carvedilol. Increased possibility of hypotension and bradycardia
Loratadine-ipratropium bromide	2.3	Pharmacodynamic	Ipratropium may enhance the anticholinergic effect of loratadine. Increased possibility of tachycardia, constipation, urinary retention, pupil dilation, vasodilation, and hyperthermia
Furosemide-potassium chloride	1.5	Pharmacodynamic	Potassium chloride increases and furosemide decreases serum potassium. Net effect of interaction is not clear

Factors Associated With Potential Drug-Drug Interactions

In univariate logistic regression analysis, age (odds ratio (OR): 1.04, 95% CI: 1.0-1.07, p = 0.038), Arab nationality (OR: 3.46, 95% CI: 0.48-9.24, p = 0.044), more than four comorbidities (OR: 5.46, 95% CI: 1.65-18.08, p = 0.005), and polypharmacy (OR: 39.96, 95% CI: 9.72-164.25, p < 0.001) were significantly associated with occurrence of pDDIs. However, the results of multivariate logistic regression analysis revealed that Arab nationality (OR: 14.21, 95% CI: 0.38-49.74, p = 0.015) and polypharmacy (OR: 62.34, 95% CI: 7.97-487.64, p < 0.001) were the independent predictors of occurrence of pDDIs in our study population (Table 3).

**Table 3:** Univariate and multivariate analyses of factors associated with the occurrence of potential drug-drug interactions in CKD patients. Polypharmacy is defined as the intake of five or more medications per day. OR: odds ratio; CI: confidence interval; CKD: chronic kidney disease

Variable (reference)	Univariate model		Multivariate model	
	OR (95% CI)	P-value	OR (95% CI)	P-value
Age, years	1.04 (1.00-1.07)	0.038	1.01 (0.96-1.06)	0.651
Gender (Male)				
Female	1.57 (0.50-4.96)	0.444	0.87 (0.17-4.45)	0.873
Nationality (Non-Arab)				
Arab	3.46 (0.48-9.24)	0.044	14.21 (0.38-49.74)	0.015
CKD stage (Stages 1–4)				
Stage 5	1.30 (0.33-5.12)	0.699	0.69 (0.06-7.61)	0.766
Length of hospital stay, days	1.02 (0.96-1.12)	0.770	1.01 (0.88-1.16)	0.880
Number of comorbidities (≤4)				
>4	5.46 (1.65-18.08)	0.005	3.01 (0.44-20.19)	0.257
Polypharmacy (<5 drugs)				
≥5 drugs	39.96 (9.72-164.25)	<0.001	62.34 (7.97-487.64)	<0.001
Type of comorbidities (No)				
Diabetes	2.74 (0.89-8.45)	0.079	3.30 (0.66-16.40)	0.144
Anemia	0.80 (0.16-3.85)	0.781	1.16 (0.08-16.53)	0.913
Hyperphosphatemia	1.48 (0.37-5.85)	0.572	0.23 (0.01-3.55)	0.291

Furthermore, negative binomial regression analysis was performed to establish the association between the number of pDDIs and different factors associated with CKD patients. The results revealed that the probability of occurrence of a pDDI increased 2.7 folds (IRR: 2.7, 95% CI: 2.09-3.48, p < 0.001) in the presence of dyslipidemia and 1.2 folds in the presence of diabetes mellitus (IRR: 1.2, 95% CI: 1.01-1.54, p = 0.040) as comorbid conditions. With every one-day increase in the length of hospital stay, the likelihood of pDDIs increased 1.02 times (IRR: 1.01, 95% CI: 1.00-1.03, p = 0.015) and polypharmacy (five or more medications) increased the likelihood of pDDIs by 6.3 times (IRR: 6.30, 95% CI: 3.04-13.02, p < 0.001) compared to no polypharmacy (Table 4).

**Table 4:** Negative binomial regression model demonstrating the association between the number of potential drug-drug interactions and different variables in CKD patients. Polypharmacy is defined as the intake of five or more medications per day. IRR: incidence rate ratio; CI: confidence interval

Variable (reference)	IRR	95% CI	P-value
Age, years	1.0	0.99-1.01	0.223
Gender (Male)			
Female	0.94	0.80-1.09	0.425
Nationality (Non-Arab)			
Arab	1.22	0.97-1.52	0.080
CKD stage (Stage 2 and 3)			
Stage 4	0.73	0.50-1.07	0.106
Stage 5	0.87	0.67-1.14	0.332
Length of hospital stay, days	1.02	1.0-1.03	0.015
Number of comorbidities (≤4)			
>4	1.02	0.73-1.41	0.908
Type of comorbidities (No)			
Hypertension	0.76	0.41-1.41	0.385
Diabetes	1.24	1.01-1.54	0.040
Anemia	1.01	0.82-1.25	0.879
Dyslipidemia	2.70	2.09-3.48	<0.001
Hyperphosphatemia	0.98	0.77-1.25	0.922
Polypharmacy (<5)			
≥5	6.30	3.04-13.02	<0.001

Adverse Drug Reactions

During the study period, a total of 14 ADRs were identified in 14 patients. Hypoglycemia (n = 3, 21.4%) and hyperkalemia (n = 3, 21.4%) were the most common suspected ADRs, followed by hypokalemia, elevated liver enzyme, and hyponatremia (n = 14.3% each). The spectrum of suspected ADRs and associated drugs is represented in Table 5. Furosemide was the most common drug involved in ADRs (n = 4, 28.5%), followed by linagliptin and valsartan (n = 3, 21.4%).

**Table 5:** Suspected adverse drug reactions and associated drugs. ADRs: adverse drug reactions

ADRs	Associated drugs	n (%)
Edema	Amlodipine	1 (7.1)
Dizziness	Moxonidine	1 (7.1)
Hypokalemia	Furosemide	2 (14.3)
Elevated liver enzymes	Atorvastatin	2 (14.3)
Hyponatremia	Furosemide	2 (14.3)
Hypoglycemia	Linagliptin	3 (21.4)
Hyperkalemia	Valsartan	3 (21.4)

Causality Assessment of Suspected Adverse Drug Reactions

**Figure 2:**
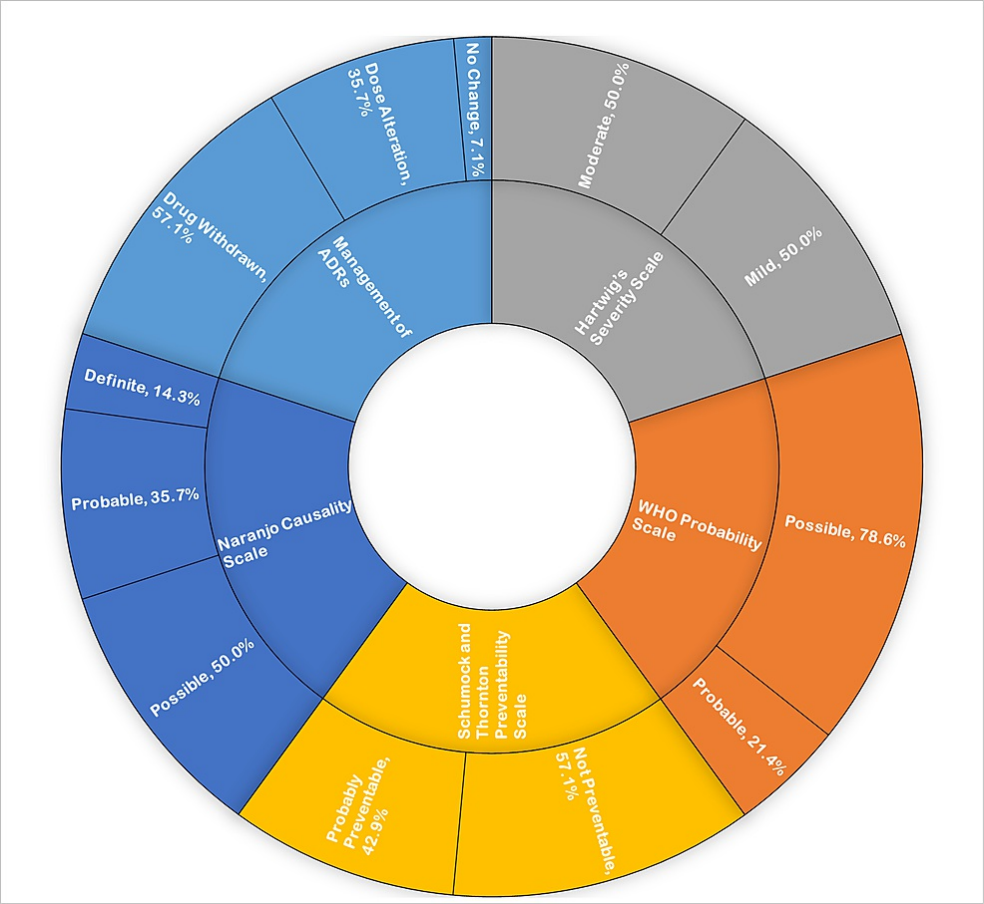
Causality assessment and management of suspected adverse drug reactions. ADRs: adverse drug reactions; WHO: World Health Organization

According to the Naranjo causality scale, the majority of suspected ADRs were possible (n = 7, 50.0%), followed by probable (n = 5, 35.7%), and definite (n = 2, 14.3%). Based on the World Health Organization probability scale, there were 11 (78.6%) possible and three (21.4%) probable ADRs. Severity assessment using Hartwig’s severity scale categorized seven (50.0%) ADRs as moderate and the remaining seven (50.0%) as mild. Preventability assessment using modified Schumock and Thornton scale indicated that eight (57.1%) ADRs were not preventable and six (42.9%) were probably preventable (Figure 2).

Management of Suspected Adverse Drug Reactions

For the management of ADRs, the suspected drug was withdrawn in 57.1% of cases, dose alteration was done in 35.7% of cases, and no change in the prescription was done in 7.1% of cases (Figure 2).

## Discussion

The present study sheds light on two common drug-related problems, DDIs and ADRs, among CKD patients. The risk of drug-related problems in CKD patients is high because of increased medication burden and impaired renal function. The estimated prevalence of DDIs in CKD patients ranges from 56.9% to 89.1% [8]. The present study reports a high prevalence of pDDIs (89.2%) in patients with CKD. A similar finding was reported in a study conducted by Al-Ramahi and colleagues in which the prevalence of pDDIs among 275 hemodialysis patients was found to be 89.1% [21]. Another study conducted by Chaudhary and colleagues reported the prevalence of pDDIs to be 78.3% [22]. These findings can be attributed to the fact that majority of the CKD patients in the study (116 out of 130) were prescribed more than five drugs, with a mean number of drugs prescribed per patient being 11.1 ± 3.8. High medication burden in CKD patients has been linked to increased prevalence of pDDIs [11,23].

The majority of the patients (46.2%) had 1-5 interactions, followed by 6-10 interactions (30.0%), with mean pDDIs per patient of 5.4 ± 4.4. Similar pDDI distribution among CKD patients has been reported by previous studies [21,24]. Different studies have employed different drug interaction checkers such as Micromedex, Medscape, LexiComp, and DrugReax, each with its own identification and classification of DDIs. PEPID 12.1 drug interaction checker was used to analyze pDDIs in this study. PEPID grades DDIs based on the level of concern as non-significant, minor, moderate, significant, and life-threatening. Most pDDIs (70.7%) identified were of moderate level of concern. Moderate DDIs may require monitoring of drug therapy and, if required, dose adjustments depending on patient response [16]. Previous investigations on cohorts of CKD patients found that majority of DDIs were of the moderate type, followed by minor or mild and significant type. Major or life-threatening DDIs accounted only for 0.1-1% of all observed DDIs [24].

Univariate logistic regression analysis revealed that age, higher number of comorbidities, and polypharmacy were significantly associated with occurrence of pDDIs in the study population. These findings are in agreement with a study conducted by Al-Ramahi and colleagues [21] in which higher age, higher number of comorbid conditions, and more than five medications were significantly associated with the number of potential interactions. Multivariate logistic regression identified polypharmacy as an independent predictor of the occurrence of pDDIs. These findings can be attributed to the multimorbid aged CKD patients in advanced stage of the disease requiring management with complex therapeutic regimens consisting of multiple drugs. Likewise, previous studies have also found polypharmacy to be significantly associated with drug-related problems [25] such as DDIs and ADRs [26]. Additionally, Arab nationality was also independently associated with occurrence of pDDIs that may be attributed to older patients with multiple comorbidities and advanced disease stage in this group.

Considering the high prevalence of polypharmacy and pDDIs in the study population, we followed a linear approach and performed a negative binomial regression analysis to examine the association between the number of pDDIs and different factors associated with CKD patients. The results revealed that comorbid conditions such as dyslipidemia and diabetes increased the probability of occurrence of a pDDI by 2.7 and 1.2 folds, respectively. Furthermore, the likelihood of pDDIs increased with every one-day increase in the length of hospital stay by 1.02 times and polypharmacy increased the likelihood of pDDIs by 6.3 times. These results fortify the fact that comorbid conditions and high medication burden increase the number of potential interactions.

CKD is a well-documented risk factor for the occurrence of ADRs. Elderly CKD patients are at higher risk of developing ADRs due to multimorbidity, polypharmacy, and age, as well as CKD-related variations in drug pharmacokinetics and pharmacodynamics [9]. The incidence of ADRs in the present study was 10.7%, which is lower than the findings of a study conducted in India that reported an overall ADR incidence of 12.9% in its CKD population [27]. However, our reported ADR incidence was similar to a study conducted in the UAE in which during the hospital stay, 12.1% of CKD patients experienced a definite or probable ADR [28].

Angiotensin receptor blockers (valsartan), dipeptidyl peptidase-4 inhibitors (linagliptin), and diuretics (furosemide) were the most common drugs implicated in ADRs in our study. Previous studies conducted in CKD patients also reported involvement of these medication classes in the occurrence of ADRs [9,28]. Hyperkalemia, hypoglycemia, and hypokalemia were the most common ADRs reported in the study population. These findings may be attributed to the fact that valsartan, linagliptin, and furosemide were common medications implicated in these ADRs, which are known to cause hyperkalemia, hypoglycemia, and hypokalemia, respectively.

Regarding drug-related causality, the majority of the ADRs in the study were possible, followed by probable and definite. Similar causality pattern was reported by a study conducted in a tertiary care teaching hospital in India in which the majority of the ADRs (57.7%) were classified as possible, followed by probable (42.2%) [27]. Moreover, the ADRs observed in our study were of mild and moderate severity, which is in line with the severity assessment of ADRs reported by a study among CKD patients hospitalized in a public healthcare center [29]. Remedial action was taken for the appropriate management of the observed ADRs. These remedial actions included withdrawing the suspected drug in most cases, altering the dose in one-third of the cases, and no change for the remaining cases.

Identification and evaluation of drug-related problems, such as DDIs and ADRs, in complex CKD population is a challenging task; nevertheless, it is very important to assess the outcomes and their impact on these vulnerable patients. Early identification, timely management, and resolution of these drug-related problems can play a vital role in minimizing the associated morbidity and mortality.

There were certain limitations to our study. First, its single-centered design with a small sample size restricts the generalizability of the results. Second, the study site was a government hospital and its results may vary from hospitals in different geographical areas with other specialties. Third, the cross-sectional nature of the study limits the causal inference of the identified relationships. Fourth, pDDIs reported in this study were discovered theoretically and may not present clinically.

## Conclusions

This study investigated two important drug related problems, pDDIs and ADRs, in CKD population. High proportion of CKD patients in the study had pDDIs. The level of concern for most pDDIs was mild to moderate. Polypharmacy was an independent predictor of occurrence of pDDIs. Length of hospital stay and comorbid conditions, such as dyslipidemia and diabetes mellitus, were significantly associated with increased likelihood of pDDIs. Furthermore, there was a burden of ADRs in the study population, of which most ADRs were possible and of mild to moderate severity.

High prevalence of drug-related problems in CKD patients is a significant challenge as it influences morbidity, mortality, and quality of life of these patients. Prevention, identification, and resolution of these problems can play a vital role in achieving better clinical outcomes, which can be achieved through medication optimization. Medication optimization in CKD patients requires a proactive interdisciplinary collaboration between clinicians, clinical pharmacists, and other healthcare professionals.
